# Immunolocalization of an Amino-Terminal Fragment of Apolipoprotein E in the Pick's Disease Brain

**DOI:** 10.1371/journal.pone.0080180

**Published:** 2013-12-02

**Authors:** Troy T. Rohn, Ryan J. Day, Lindsey W. Catlin, Raquel J. Brown, Alexander J. Rajic, Wayne W. Poon

**Affiliations:** 1 Department of Biological Sciences, Boise State University, Boise, Idaho, United States of America; 2 Institute for Memory Impairments and Neurological Disorders, University of California Irvine, Irvine, California, United States of America; University of South Florida Alzheimer's Institute, United States of America

## Abstract

Although the risk factor for apolipoprotein E (apoE) polymorphism in Alzheimer's disease (AD) has been well described, the role that apoE plays in other neurodegenerative diseases, including Pick's disease, is not well established. To examine a possible role of apoE in Pick's disease, an immunohistochemical analysis was performed utilizing a novel site-directed antibody that is specific for an amino-terminal fragment of apoE. Application of this antibody, termed the amino-terminal apoE cleavage fragment (nApoECF) antibody, consistently labeled Pick bodies within area CA1 of the hippocampus in 4 of the 5 cases examined. Co-localization of the nApoECF antibody with PHF-1, a general marker for Pick bodies, as well as with an antibody to caspase-cleaved tau (TauC3) was evident within the hippocampus. While staining of the nApoECF antibody was robust in area CA1, little co-localization with PHF-1 in Pick bodies within the dentate gyrus was observed. A quantitative analysis indicated that approximately 86% of the Pick bodies identified in area CA1 labeled with the nApoECF antibody. The presence of truncated apoE within Pick bodies suggests a broader role of apoE beyond AD and raises the question as to whether this protein contributes to pathogenesis associated with Pick's disease.

## Introduction

Human apoE is polymorphic with three major isoforms, apoE2, E3, and E4, which differ by single amino acid substitutions at positions 112 and 158 [1]. Inheritance of one copy of the *APOE4* allele increases AD disease risk fourfold, while two copies raises the risk tenfold [2]. Structurally, apoE4 is a 34 kDa protein composed of 299 amino acids and contains two major domains, referred to as the N-terminal (∼20 kDa) and C-terminal (∼10 kDa) domains, which are connected by a short hinge region [3]. How apoE4 confers disease risk in AD is unknown, but emerging evidence suggests that proteolytic cleavage of apoE4 may lead to a loss or toxic-gain of function thus contributing to disease pathogenesis (for review see [4]). Several studies have identified the presence of ∼18 kDa band in human AD brain extracts, suggesting cleavage of apoE4 near position D172 [5], [6]. Previous studies have shown that apoE4 is highly susceptible to proteolysis compared to apoE3, and apoE4 fragments (14–20 kDa) have been identified in the AD brain [5]. To determine if this site within apoE is cleaved by proteases in the AD brain, we developed and characterized a site-directed neoepitope antibody directed towards the amino-terminal fragment that would be generated following cleavage at D172. Application of this antibody, *in situ*, revealed specific localization within NFTs that co-localized with PHF-1 in the AD brain with a preference of localization in those AD cases with either the 3/4 or 4/4 *APOE* genotypes [7]. The purpose of the present study was to examine whether amino-terminal fragments of apoE can be documented in Pick's disease.

Pick's disease is classified as a tauopathy and is characterized by filamentous neuronal and glial hyperphosphorylated tau [8]. Pick's disease is associated with severe neuronal and glial loss leading to frontotemporal lobe atrophy [8]. Pathologically, a key feature of Pick's disease is the presence of Pick bodies that are composed of aggregates of hyperphosphorylated tau [9]. Clinically, Pick's disease is characterized by loss of verbal skills and progressive dementia [10]. *APOE* gene polymorphism is known to be associated with Pick's disease [11]–[13]. Despite this known association between apoE and Pick's disease, few studies have examined apoE immunoreactivity in the Pick's disease brain with the exception of Hayashi et al., who demonstrated the presence of apoE immunoreactivity in Pick bodies from two cases with the *APOE* genotypes of 3/4 and 3/3, respectively [14]. To determine if apoE is cleaved in Pick's disease, we analyzed five cases by immunohistochemistry using our novel, in house antibody that detects amino-terminal cleavage fragments of apoE (termed nApoECF antibody). We found strong immunolabeling of this antibody within Pick bodies in the CA1 region of four of five cases examined. In addition, co-localization of the nApoECF antibody with PHF-1 and an antibody to caspase-cleaved tau (TauC3) was observed. Taken together, these results demonstrate the presence of apoE amino-terminal fragments in the Pick's disease brain. The high degree of co-localization between PHF-1, caspase-cleaved tau, and our nApoECF antibody suggest a potential causal relationship between modified tau and cleaved apoE.

## Results

Previous characterization of the nApoECF antibody indicated that it is highly specific for an 18 kDa amino-terminal fragment of apoE [7]. This in house antibody was synthesized based upon a putative caspase-cleavage site (DADD) at position D172 of the full-length protein. Application of this antibody to AD frontal cortex brain sections revealed specific localization within neurofibrillary tangles (NFTs) that was dependent upon the *APOE* genotype: 4/4≥3/4>3/3 [7]. However, *in vitro* cleavage of apoE4 by caspase-3 to generate an 18 kDa fragment detectable by the nApoECF antibody was unsuccessful [7]. To determine if amino-terminal fragments of apoE can be detected in Pick's disease, an immunohistochemical study utilizing the nApoECF antibody was performed utilizing fixed hippocampal brain sections from five Pick cases. Case demographics for the Pick cases used in this study are presented in **Table 1**. Notice that the *APOE* genotype was confirmed in 3/5 cases. All five cases had a primary neuropathological diagnosis of Pick's disease.

**Table 1:** Case Demographics.

Case	Age	Sex	PMI	ApoE Genotype	NPD	Braak and Braak	Plaque Stage
**1**	66	F	3	N/A	PiD	Stage 0	None
**2**	78	M	15.37	N/A	PiD	Stage 0	None
**3**	63	F	6.47	3/3	PiD	Stage 0	None
**4**	72	M	4.5	3/3	PiD	Stage 0	None
**5**	63	F	3.8	3/3	PiD	Stage 0	None
**6**	73	F	5.1	3/3	Normal	Stage 0	None
**7**	75	F	2.75	N/A	Normal	Stage 0	None

PMI, postmortem interval in hours; NPD, neuropathological diagnosis.

**Figure 1:**
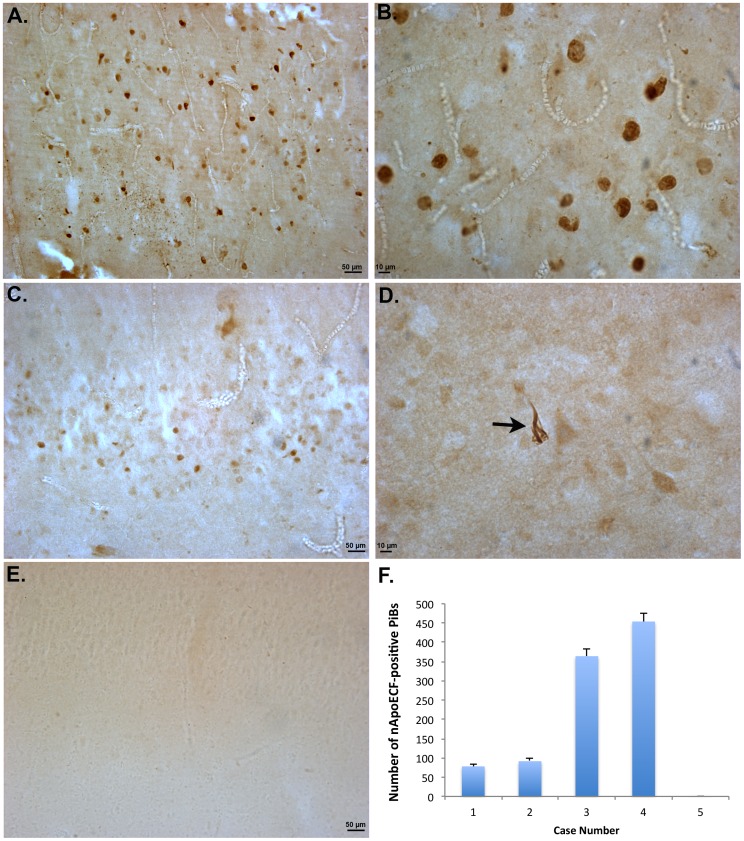
Localization of an amino-terminal fragment of apoE in Pick's disease. (**A and B**): Low (A) and high magnification (B) of representative labeling from a Pick's case (Case #4, Table 1) utilizing the nApoECF antibody illustrating staining in the CA1 region of the hippocampus within Pick bodies. (**C**): Representative staining in the dentate gyrus (Case #3, Table 1) showing weaker, sparser labeling with the nApoECF antibody. (**D**): Representative staining in case #5 (Table 1), which had a primary neuropathological diagnosis of Pick's disease showing a complete lack of labeling of Pick bodies but instead labeling of apparent NFTs (arrow). (**E**): Representative staining of the nApoECF antibody in an age-matched control case revealing a complete absence of labeling. (**F**): Quantitative analysis of the number of Pick bodies that were nApoECF-positive for each Pick case examined with the nApoECF antibody. Data are the average of two independent counts ±S.D. For case demographics please see **Table 1**.

As an initial step, we screened all five cases for nApoECF immunoreactivity using bright-field microscopy. Following application of the nApoECF antibody, strong labeling of Pick bodies within area CA1 was observed in 4/5 cases (Fig. 1A and B). A much weaker staining pattern of Pick bodies was also observed in granule cells of the dentate gyrus (Fig. 1C). Strong immunolabeling within area CA1 was in contrast to age-matched control subjects, where there was a complete lack of staining within the hippocampus (Fig. 1E). In one case (Case #5, **Table 1**) we did not observe any labeling of the nApoECF antibody within Pick bodies, but instead documented the occasional staining of apparent NFTs (arrow, Fig. 1D). Of interest was additional screening of this case with PHF-1, a well-known marker for Pick bodies, revealed the complete absence of any Pick bodies in the hippocampus for this case. Further analysis of this case by the *Institute for Memory Impairments and Neurological Disorders at the University of California, Irvine* confirmed the lack of Pick bodies within hippocampal tissue sections. Interesting, this case did have a secondary neuropathological diagnosis of frontal temporal dementia. In any event, the specificity of the nApoECF antibody appeared to be confined within Pick bodies or NFTs as no other labeling was observed in any other cell type including glial cells. A quantitative analysis of the number of nApoECF-positive Pick bodies in area CA1 indicated a wide variability between the five cases and was highest in two cases that were listed as having an *APOE* genotype of 3/3 and in which the presence of Pick bodies was confirmed (Fig. 1F). Previous studies have shown that *in vitro*, apoE4 is highly susceptible to proteolysis compared to apoE3 [5]. However, because this fragment was observed in PiD cases harboring the *APOE* 3/3 genotype, these results suggest that this isoform is also capable of undergoing fragmentation *in vivo*.

**Figure 2:**
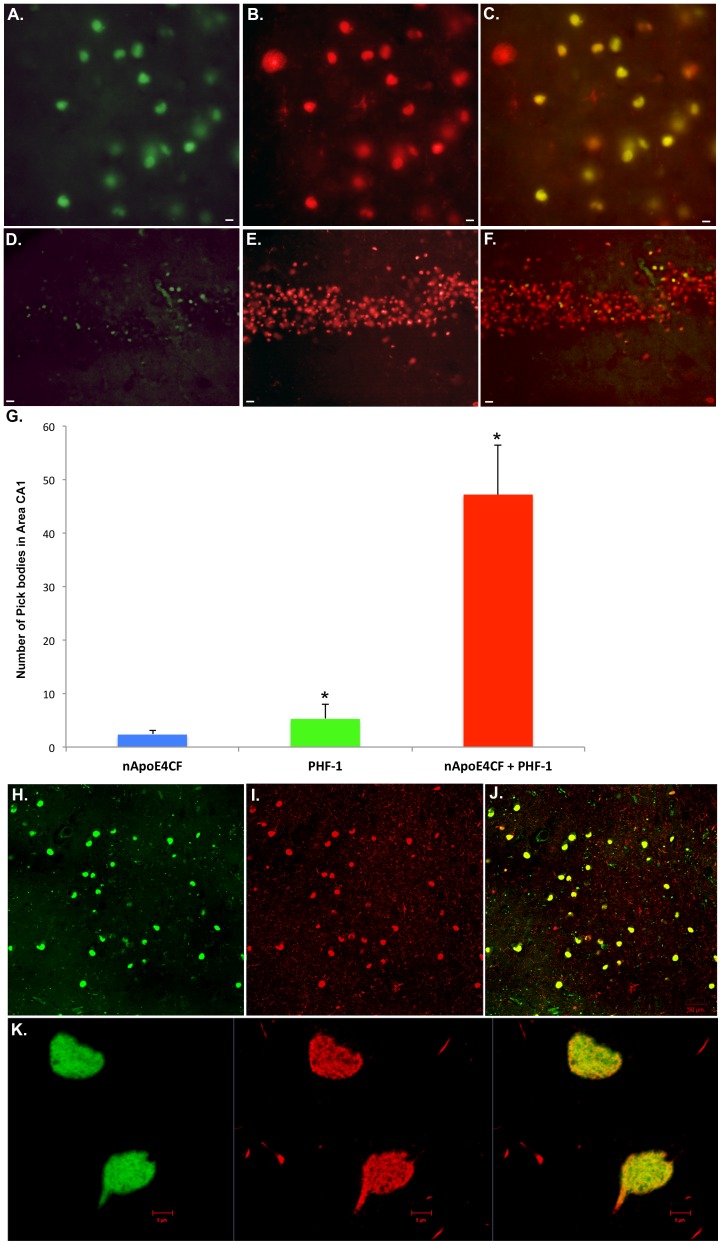
Labeling of nApoECF antibody within the hippocampus co-localizes with PHF-1 and is regionally defined. (**A–C**): Representative immunofluorescence double-labeling in Case #3 (Table 1) utilizing the nApoECF antibody (green, Panel A) and PHF-1 (red, Panel B) revealed strong co-localization of the two antibodies within Pick bodies of area CA1 (Panel C). (**D–F**): In contrast to area CA1, strong immunofluorescence was detected using PHF-1 (red, Panel E), but only weak labeling was observed within granule cells of the dentate gyrus following application of the nApoECF antibody (green, Panel D). Panel F shows the overlap image with yellow indicating Pick bodies double-labeled with both antibodies. (**G**): Quantification of Pick bodies double-labeled by PHF-1 and nApoECF. Data show the number of Pick bodies labeled with nApoECF alone (blue bar), PHF-1 alone (green bar) or those Pick bodies that were labeled with both antibodies (red bar) in area CA1. Pick bodies were identified in a 20× field within area CA1 by immunofluorescence overlap microscopy (n = 3 fields for 4 different Pick cases) ±S.D., *p<0.05. Data indicated that roughly 86% of all identified Pick bodies within area CA1 were labeled with both antibodies. (**H–J**): Double-labeled confocal immunofluorescence images with the nApoECF antibody (green, H), PHF-1 (red, I), and the two images overlapped (J). All scale bars represent 10 µm, except for confocal images in Panel H–J, which represent 50 µm. (**K**): High magnification confocal overlapped image representing nApoECF (green channel, left), PHF-1 (red channel, middle), and overlapped image (yellow/orange, right); notice filamentous nature of staining within the two Pick bodies. Scale bars for Panel K represent 5 µm.

To determine the extent of localization of the nApoECF antibody within Pick bodies, double-label immunofluorescence studies were undertaken using PHF-1 as a marker for Pick bodies. As shown using conventional immunofluorescence microscopy, strong co-localization of the two antibodies within Pick bodies was observed in area CA1 in Pick cases (Fig. 2A–C). The yellow/orange color in Panel C depicts the co-localization between the two markers. Confocal analysis confirmed the colocalization of the nApoECF antibody with PHF-1 within Pick bodies of area CA1 (Fig. 2H–J) and at high magnification, the filamentous nature of PHF-1 labeling was evident within Pick bodies (Fig. 2K).

As with the results utilizing bright-field, we only observed weak labeling of the nApoECF antibody within Pick bodies located in the dentate gyrus (Fig. 2D). This was in contrast to PHF-1, which in addition to labeling Pick bodies in area CA1, also displayed strong and widespread labeling of Pick bodies throughout granule cells of the dentate gyrus (Fig. 2E and F). Therefore, based on these results, fragmentation of apoE displayed a regional pattern of distribution being predominantly located within the CA1 region of the hippocampus of Pick cases. A quantitative analysis indicated that within the CA1 region, 86% of identified Pick bodies using PHF-1 were also labeled with the nApoECF antibody (Fig. 2G). These results suggest that whatever aberrant processes are responsible for generating modified tau; similar pathways may also be involved in the fragmentation of apoE.

To determine any possible correlation between the various groups, Pearson coefficients were determined. The Pearson coefficient comparing nApoECF labeling alone to those Pick bodies labeled with both PHF-1 and nApoECF was 0.23; for PHF-1 alone versus nApoECF alone, −0.10; and for nApoECF and PHF-1 versus PHF-1 alone, −0.62. These data suggest a moderate negative correlation between the number of Pick bodies labeled with PHF-1 alone and those labeled with both PHF-1 and nApoECF.

**Figure 3:**
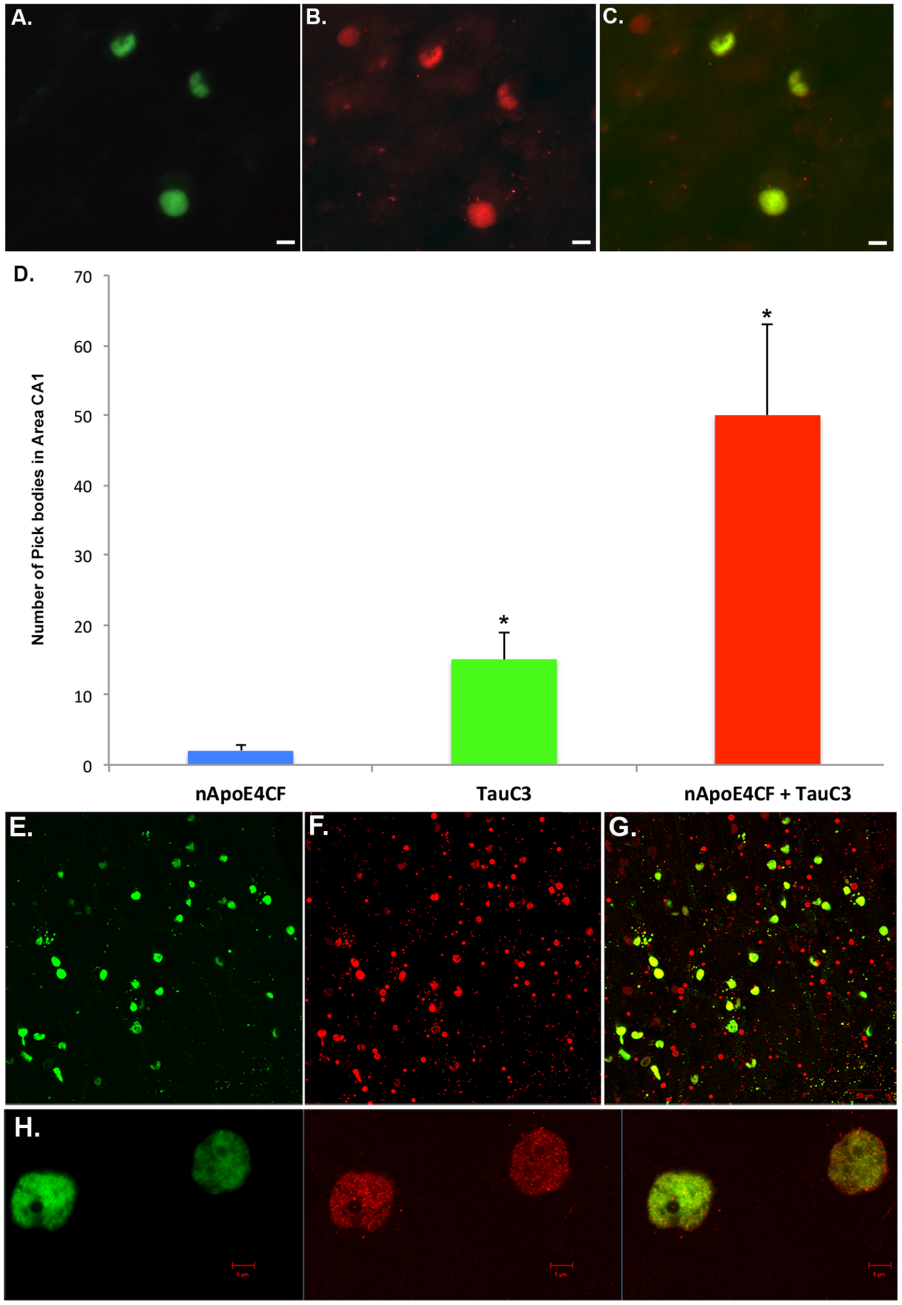
Co-localization of the nApoECF antibody with caspase-cleaved tau. (**A–C**): Representative immunofluorescence double-labeling in Case #4 (Table 1) within the CA1 region of the hippocampus in Pick's disease utilizing the nApoECF antibody (green, Panel A), caspase-cleaved tau (red, Panel B) and with the overlap image shown in Panel C. All scale bars represent 10 µm. (**D**): Quantification of Pick bodies double-labeled by TauC3 (caspase-cleaved tau) and nApoECF in area CA1 of the hippocampus. Data show the number of Pick bodies labeled with nApoECF alone (blue bar), Tauc3 alone (green bar) or those Pick bodies that were labeled with both antibodies (red bar). Pick bodies were identified in a 20× field within area CA1 by immunofluorescence overlap microscopy (n = 3 fields for 4 different Pick cases) ±S.D., *p<0.05. Data indicated that roughly 75% of all labeled Pick bodies within area CA1 were co-localized with both antibodies. (**E–G**): Double-labeled confocal immunofluorescence images with the nApoECF antibody (green, E), PHF-1 (red, F), and the two images overlapped (G). (**H**): High magnification confocal overlapped image representing nApoECF (green channel, left), TauC3 (red channel, middle), and overlapped image (yellow/orange, right). Scale bars in Panel H represent 5 µm.

In a previous report, we documented the presence of caspase-cleaved TAR DNA-binding protein (TDP)-43 and caspase-cleaved tau in Pick's disease that revealed an almost identical staining pattern as observed with the nApoECF antibody in the present study. Caspase-cleaved TDP-43 and tau was found predominantly within Pick bodies of area CA1, with little staining found in granule cells of the dentate gyrus [15]. Therefore, we determined the extent of co-localization of the nApoECF antibody with caspase-cleaved tau by performing double-label immunofluorescence experiments utilizing an antibody specific for caspase-cleaved tau (TauC3) [16]. Co-localization between the TauC3 and nApoECF antibodies was observed in area CA1, with 75% of identified Pick bodies containing caspase-cleaved tau and the amino-terminal fragment of apoE (Fig. 3A–D). Co-localization between TauC3 and the nApoECF antibody was also confirmed following confocal double-label immunofluorescence experiments (Fig. 3E–G). Interesting at high magnification, confocal analysis revealed the punctate nature of the labeling the TauC3 antibody (Panel H).

To determine any possible correlation between the various groups, Pearson coefficients were determined. The Pearson coefficient comparing nApoECF labeling alone to those Pick bodies labeled with both TauC3 and nApoECF was −0.15; for TauC3 alone versus nApoECF alone, 0.017; and for nApoECF and PHF-1 versus PHF-1 alone, −0.15. These data suggest a lack of correlation between any of the groups tested.

## Discussion

Harboring the *APOE4* allele enhances the risk for AD [2] and several reports have suggested that it is the proteolytic cleavage of apoE4 into neurotoxic N- and C-terminal fragments that may provide one mechanism by which this protein contributes to AD pathogenesis (for recent review see [4]). ApoE4 is highly susceptible to proteolysis compared to apoE3, and apoE4 fragments (14–20 kDa) have been identified in the AD brain [5]. However, whether apoE4 or other isoforms of apoE play any role in the etiology of Pick's disease is currently unknown. Pick's disease is classified as a tauopathy that is characterized pathologically by the presence of round Pick body structures containing hyperphosphorylated, aggregated tau [17]. Despite that *APOE* gene polymorphism is known to be associated with Pick's disease [11]–[13], very few studies have examined a potential role of apoE in Pick's disease with the exception of Hayashi et al., who demonstrated the presence of apoE immunoreactivity in Pick bodies from two cases with the *APOE* genotypes of 3/4 and 3/3, respectively [14]. Therefore, we sought to determine a potential role of apoE in Pick's disease by examining for the presence of fragmented apoE utilizing an in house antibody designed to detect the amino-terminal fragment of apoE [7]. In AD this antibody, termed the amino-terminal apoE cleavage-fragment (nApoECF) antibody, predominantly labeled NFTs in the AD brain [7]. Moreover, not only did we detect nApoECF-positive NFTs in 3/4 and 4/4 *APOE* genotypes, we also observed limited labeling of NFTs in *APOE* 3/3 genotype cases. To perform an immunohistochemical analysis utilizing the nApoECF antibody, we examined 5 available cases of Pick's disease in which the *APOE* genotype of 3/3 was identified in three of the five cases, while the *APOE* genotype of the other two cases was not available (**Table 1**). We predicted at the outset that we would observe very little staining with the nApoECF antibody in those 3/3 cases based on our previous findings in the AD brain [7], but instead observed nApoECF staining within Pick bodies in two *APOE* 3/3 cases, with the third case (Case #5) being the exception. However, further analysis of Case #5 indicated that this case in fact did not harbor any Pick bodies based on the lack of PHF-1 labeling and may have instead, represented a FTD case. To our knowledge, this is the first demonstration of fragmentation of apoE in the Pick's disease brain and moreover, the fragmentation occurred in cases with the *APOE* genotype of 3/3.

An interesting feature of the nApoECF labeling was the regional distribution of staining within Pick bodies specifically in area CA1 of the hippocampus. This staining pattern was in contrast to PHF-1, which revealed widespread labeling of Pick bodies in both area CA1 and also within granule cells of the dentate gyrus. This regional staining pattern was very reminiscent of the regional distribution pattern we previously documented for caspase-cleaved tau and caspase-cleaved TDP-43 [15]. Therefore, we performed double-label immunofluorescence experiments to determine the extent of co-localization between TauC3 and the nApoECF antibody. Strong co-localization between these two antibodies was observed in over 75% of identified Pick bodies in area CA1. These data suggest that the same processes that lead to aberrant cleavage of tau may also be present to facilitate the cleavage of apoE. However, it is important to note that the protease responsible for cleaving apoE into an 18 kDa fragment that is detected by the nApoECF antibody remains unknown. *In vitro*, we have previously determined that it is not caspase-3 [7].

In summary, we have documented the presence of amino-terminal fragments of apoE3 within Pick bodies that displayed a regional localization, and showed a high degree of co-localization with PHF-1 and caspase-cleaved tau. These results suggest that apoE and its' cleavage is not restricted to AD, but may be present in other neurodegenerative diseases. Because apoE N-terminal fragments have been shown to be neurotoxic and can promote the pathology associated with AD, it is interesting to speculate on whether these fragments found in Pick bodies contribute anyway to disease pathogenesis. Further studies examining the role of apoE, tau and any relation to specific proteases should shed light on whether apoE also contributes to disease pathogenesis in Pick's disease.

## Materials and Methods

### Immunohistochemistry

Autopsy brain tissue from five neuropathologically confirmed Pick cases were studied. Case demographics are presented in **Table 1**. Fixed hippocampal tissue sections used in this study were provided by the *Institute for Memory Impairments and Neurological Disorders at the University of California, Irvine*. Free-floating 40 µm-thick sections were used for immunohistochemical studies as previously described [18]. No approval from Boise State University Institutional Review Board was obtained due to the exemption granted that all tissue sections were fixed and received from University of California, Irvine. Sections from the hippocampus were selected for immunohistochemical analysis.

For single labeling, all sections were washed with 0.1 M Tris-buffered saline (TBS), pH 7.4, and then pretreated with 3% hydrogen peroxide in 10% methanol to block endogenous peroxidase activity. Sections were subsequently washed in TBS with 0.1% Triton X-100 (TBS-A) and then blocked for thirty minutes in TBS-A with 3% bovine serum albumin (TBS-B). Sections were further incubated overnight at room temperature with the nApoECF antibody (1∶100). Following two washes with TBS-A and a wash in TBS-B, sections were incubated in anti-rabbit or mouse biotinylated anti-IgG (1 hour) and then in avidin biotin complex (1 hour) (ABC, Elite Immunoperoxidase, Vector Laboratories, Burlingame, CA, USA). The primary antibody was visualized using brown DAB substrate (Vector Laboratories).

### Immunofluorescence Microscopy

Immunofluorescence studies were performed by incubating sections with primary antibodies overnight at a room temperature, followed by secondary anti-rabbit or mouse biotinylated anti-IgG (1 hour) and then in ABC (1 hour). Primary antibodies utilized included the nApoECF (1∶100), PHF-1 (mouse monoclonal, 1∶1,000) and TauC3 (mouse monoclonal, 1∶100). The TauC3 antibody was purchased from Millipore (Billerica, MA), while PHF-1 was a generous gift from Dr. Peter Davies (Albert Einstein College of Medicine, Bronx, NY). For immunofluorescence co-localization studies, antigen visualization was accomplished using an Alexa fluor 488-labeled tyramide (green, Ex/Em = 495/519) for one label and streptavidin Alexa fluor 555 (red, Ex/Em = 555/565) for the second label, both from Invitrogen (Carlsbad, CA). For microscopic observation and photomicrography of the DAB-labeled and fluorescent sections, an Olympus BX60 microscope with fluorescence capability equipped with a MagnaFire SP software system for photomicrography was employed. The fluorescent molecules were excited with a 100-W mercury lamp. Fluorescent-labeled molecules were detected using a filter set having a 460–500-nm wavelength band pass excitation filter, a 505-nm dichroic beam splitter, and a 510–560-nm band pass emission filter.

### Confocal microscopy

For confocal immunofluorescence imaging, the primary antibodies were visualized with secondary antibodies tagged with either Alexa Fluor 488 or Alexa Fluor 555 (Invitrogen, Carlsbad, CA.) Images were taken with the Zeiss LSM 510 Meta system combined with the Zeiss Axiovert Observer Z1 inverted microscope and ZEN 2009 imaging software (Carl Zeiss, Inc., Thornwood, NY). Confocal Z-stack and single plane images were acquired with an Argon (488 nm) and a HeNe (543 nm) laser source. Z-stacks images were acquired using a 20× Plan-Apochromat (NA 0.8) objective, emission band passes of 505–550 nm for the detection of the nApoECF antibody (green channel, Alexa Fluor 488) and 550–600 nm for both the detection of PHF-1 (red channel, Alexa Fluor 555) and TauC3 (red, Alexa Fluor 555). All images displayed are 2-D, maximal intensity projections generated acquired Z-stacks. Single plane images were acquired with a 63× Plan-Apochromat oil-immersion objective (NA 1.4) with emission long pass of 505 nm for the detection of the nApoECF antibody (green channel, Alexa Fluor 488) and 550–600 nm for the detection of TauC3 (red channel, Alexa Fluor 555).

### Statistical analysis

To determine the percent co-localization, a quantitative analysis was performed as described previously [7], [18] by taking 20× immunofluorescence, overlapping images from three different fields in area CA1 in four separate Pick cases. Capturing was accomplished by using a 2.5× photo eyepiece, and a Sony high resolution CCD video camera (XC-77). As an example, to determine the percent co-localization between nApoECF and PHF-1, photographs were analyzed by counting the number of nApoECF-, PHF-1-positive Pick bodies alone per 20× field for each case, and the number of cells labeled with both PHF-1 and nApoECF. Data are representative of the average number (±S.D.) of each antibody alone or co-localized with both antibodies in each 20× field (3 fields total for 4 different cases). Statistical differences in this study were determined using Student's two-tailed T-test employing Microsoft Office Excel. To determine any possible correlations between the various groups, Pearson's coefficients were determined using Microsoft Office Excel.
